
JOURNAL INFORMATION
==============================
NLM Title Abbreviation: ScientificWorldJournal
Journal ID: ScientificWorldJournal
NLM Journal ID: 101131163
Journal ID: TSWJ

The Scientific World Journal

ISSN: 2356-6140
EISSN: 1537-744X
Publisher: Wiley

ARTICLE INFORMATION
==============================
PMCID: PMC5956476
PMID: 15617199
DOI: 10.1100/tsw.2004.37
Article ID: 941279
Article version: 1
Subjects: Original Articles

Ultrasound Findings of Acute Idiopathic Scrotal Edema

Thomas A. C.
Cain Mark P. *
Casale Anthony J.
Rink Richard C.
Department of Urology, Indiana University School of Medicine, USA
*Mark P. Cain: mpcain@iupui.eduAcademic Editor: Anthony Atala

Publication date: 2004
Electronic publication date: 2004 Jun 7
Volume: 4
First page: 9
Last page: 10
Copyright: Copyright © 2004 A. C. Thomas et al.
Copyright year: 2004
License: This is an open access article distributed under the Creative Commons Attribution License, which permits unrestricted use, distribution, and reproduction in any medium, provided the original work is properly cited.
License URL: https://creativecommons.org/licenses/by/3.0/

==============================
Original Articles
*TheScientificWorldJOURNAL* (2004) 4 (S1), 9–10
ISSN 1537-744X; DOI 10.1100/tsw.2004.37

### Ultrasound Findings of Acute Idiopathic Scrotal Edema

A. Craig Thomas, Mark P. Cain*, Anthony J. Casale, and Richard C. Rink
*Department of Urology, Indiana University School of Medicine*

E-mails**:** mpcain@iupui.edu

*Previously published in the Digital Urology Journal*

**DOMAIN**: urology

Idiopathic scrotal edema is an uncommon cause of acute scrotal problems in children. This is a diagnosis that can be confirmed with color Doppler ultrasound, thus obviating the need for surgical exploration.1-4 Although the literature documents the benefit of Doppler ultrasound in identifying blood flow to the testis in these patients, there is little mentioned with regards to the scrotal wall changes on ultrasound.5-7 We submit ultrasound findings in three boys that presented to us with characteristic findings of idiopathic scrotal edema, each of which had significant scrotal wall subcutaneous edema on ultrasound examination.

All three patients presented with acute onset of scrotal swelling and erythema. Scrotal ultrasound demonstrated similar findings in all three patients, including normal appearing testicular parenchyma and excellent blood flow to both testes. There was significant peritesticular edema with the appearance of considerable fluid in the scrotal wall (see Figure 1 and 2), with marked thickening of the subcutaneous tissues. There was also a significant increase in the vascularity of the scrotal skin and subcutaneous tissues. One patient was admitted overnight for IV antibiotics due to the severity of the scrotal wall edema. Two patients received oral Benadryl for the presumed diagnosis of idiopathic scrotal edema. Recovery was uncomplicated, with complete resolution of symptoms within 48 hours in all 3 patients.

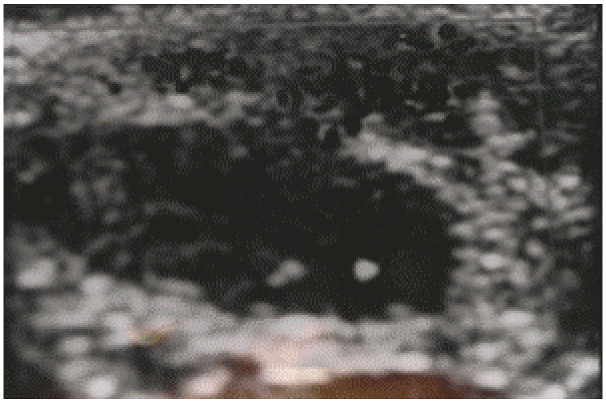

**FIGURE 1**. Scrotal Ultrasound demonstrating normal testis with significant subcutaneous edema in scrotal wall.

*Corresponding author: Riley Hospital for Children, Suite 1739, 702 Barnhill Drive, Indianapolis, IN 46202; (317)274-7446/Fax: (317)274-7481

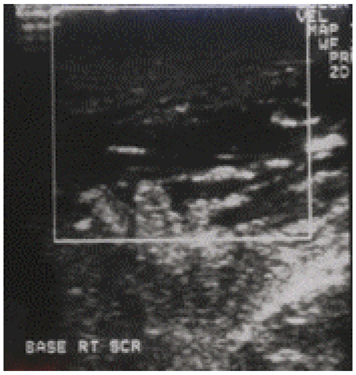

**FIGURE 2.** Patient with significant scrotal wall edema mimicking scrotal abscess.

The findings of significant scrotal wall edema on ultrasound examination of patients with idiopathic scrotal edema are most likely underreported in the literature. This is consistent with the disease process and together with findings of normal testicular blood flow, would assist in confirming the diagnosis in patients with suspected idiopathic scrotal edema, and permit conservative management with the avoidance of unnecessary scrotal exploration.

### BIBLIOGRAPHY

1. Grainger AJ, Hide, GH, Simon TE: The ultrasound appearances of scrotal oedema. European J of Ultrasound 8: 33-37, 1998.
2. Lerner RM, Mevorach RA, Hulbert WC, Rabinowitz R: Color Doppler US in the evaluation of acute scrotal disease. Radiology 176: 355-358, 1990.
3. Rabinowitz R, Hulbert WC: Acute scrotal swelling. Urologic Clinics of North America 22: 101-105, 1995.
4. Sasso F, Nucci G, Palmiotto F, et al.: Acute idiopathic scrotal oedema: rare disorder or difficult diagnosis?. International urology and nephrology 22: 475-478, 1990.
5. Melekos MD, Asbach HW, Markou SA: Etiology of acute scrotum in 100 boys with regard to age distribution. J Urol 139: 1023-1025, 1988.
6. Najmaldin A, Burge DM: Acute idiopathic scrotal oedema: incidence, manifestations and aetiology. Br J Surg 74: 634-635, 1987.
7. Brandes SB, Chelsky MJ, Hanno PM: Adult acute idiopathic scrotal edema. Urology 44: 602-605, 1994.

**This article should be referenced as follows:**

Thomas, A.C., Cain, M.P., Casale, A.J., and Rink, R.C. (2004) Ultrasound findings of acute idiopathic scrotal edema. *TheScientificWorldJOURNAL* **4 (S1)**, 9–10.

**Handling Editor:**

Anthony Atala, Principle Editor for *Urology* — a domain of *TheScientificWorldJOURNAL*.
